# Clinical Features and Evolution of Blepharospasm: A Multicenter International Cohort and Systematic Literature Review

**DOI:** 10.3389/dyst.2022.10359

**Published:** 2022-05-16

**Authors:** Laura M. Scorr, Hyun Joo Cho, Gamze Kilic-Berkmen, J. Lucas McKay, Mark Hallett, Christine Klein, Tobias Baumer, Brian D. Berman, Jeanne S. Feuerstein, Joel S. Perlmutter, Alfredo Berardelli, Gina Ferrazzano, Aparna Wagle-Shukla, Irene A. Malaty, Joseph Jankovic, Steven T. Bellows, Richard L. Barbano, Marie Vidailhet, Emmanuel Roze, Cecilia Bonnet, Abhimanyu Mahajan, Mark S. LeDoux, Victor S.C. Fung, Florence C.F. Chang, Giovanni Defazio, Tomaso Ercoli, Stewart Factor, Ted Wojno, H. A. Jinnah

**Affiliations:** 1 Department of Neurology, School of Medicine, Emory University, Atlanta, GA, United States; 2 Human Motor Control Section, National Institute of Neurological Disorders and Stroke, National Institutes of Health, Bethesda, MD, United States; 3 Department of Biomedical Informatics, School of Medicine, Emory University, Atlanta, GA, United States; 4 Department of Biomedical Engineering, Emory University and Georgia Tech, Atlanta, GA, United States; 5 Institute of Neurogenetics and Department of Neurology, University of Luebeck and University Hospital of Schleswig-Holstein, Luebeck, Germany; 6 Department of Neurology, Virginia Commonwealth University, Richmond, VA, United States; 7 Department of Neurology, University of Colorado, Aurora, CO, United States; 8 Department of Neurology, Radiology, Neuroscience, Physical Therapy and Occupational Therapy, Washington University School of Medicine, St Louis, MO, United States; 9 Department of Human Neuroscience, Sapienza University of Rome, Rome, Italy; 10 IRCCS Neuromed, Pozzilli, Italy; 11 Fixel Institute for Neurological Disease, Department of Neurology, University of Florida, Gainesville, FL, United States; 12 Parkinson’s Disease Center and Movement Disorders Clinic, Department of Neurology, Baylor College of Medicine, Houston, TX, United States; 13 Department of Neurology, University of Rochester, Rochester, NY, United States; 14 Paris Brain Institute, Inserm, CNRS, AP-HP, Salpetrière Hospital, Sorbonne University, Paris, France; 15 Rush Parkinson’s Disease and Movement Disorders Program, Department of Neurological Sciences, Rush University, Chicago, IL, United States; 16 Department of Psychology, Veracity Neuroscience LLC, University of Memphis, Memphis, TN, United States; 17 Movement Disorders Unit, Department of Neurology, Westmead Hospital and Sydney Medical School, University of Sydney, Sydney, NSW, Australia; 18 Movement Disorders Unit, Department of Neurology, Westmead Hospital, Sydney, NSW, Australia; 19 Department of Medical Sciences and Public Health, University of Cagliari, Cagliari, Italy; 20 Emory Eye Center, Emory University, Atlanta, GA, United States; 21 Department of Human Genetics, School of Medicine, Emory University, Atlanta, GA, United States

**Keywords:** Dystonia, Meige syndrome, eyes, jaw, phenotype, Blepharospasm, Oromandibular dystonia

## Abstract

**Objective:**

Blepharospasm is a type of dystonia where the diagnosis is often delayed because its varied clinical manifestations are not well recognized. The purpose of this study was to provide a comprehensive picture of its clinical features including presenting features, motor features, and non-motor features.

**Methods:**

This was a two-part study. The first part involved a systematic literature review that summarized clinical features for 10,324 cases taken from 41 prior reports. The second part involved a summary of clinical features for 884 cases enrolled in a large multicenter cohort collected by the Dystonia Coalition investigators, along with an analysis of the factors that contribute to the spread of dystonia beyond the periocular region.

**Results:**

For cases in the literature and the Dystonia Coalition, blepharospasm emerged in the 50s and was more frequent in women. Many presented with non-specific motor symptoms such as increased blinking (51.9%) or non-motor sensory features such as eye soreness or pain (38.7%), photophobia (35.5%), or dry eyes (10.7%). Non-motor psychiatric features were also common including anxiety disorders (34–40%) and depression (21–24%). Among cases presenting with blepharospasm in the Dystonia Coalition cohort, 61% experienced spread of dystonia to other regions, most commonly the oromandibular region and neck. Features associated with spread included severity of blepharospasm, family history of dystonia, depression, and anxiety.

**Conclusions:**

This study provides a comprehensive summary of motor and non-motor features of blepharospasm, along with novel insights into factors that may be responsible for its poor diagnostic recognition and natural history.

## INTRODUCTION

Dystonia is a disorder characterized by sustained or intermittent muscle contractions causing abnormal repetitive movements and postures [1]. Blepharospasm (BSP) is a subtype characterized by bilateral synchronous spasms of the orbicularis oculi and surrounding muscles [2, 3]. Additional manifestations include exaggerated blinking, eyelid fluttering, and apraxia of eyelid opening. Commonly reported non-motor features include dry or gritty sensations in the eyes, photophobia, depression, and anxiety [4-11]. BSP is particularly disabling because it interferes with vision, sometimes rendering subjects functionally blind. In approximately half of those affected, dystonia spreads from the upper face to the oromandibular area, neck, or other regions [12-17].

Idiopathic BSP has a reported prevalence between 20 and 133 cases per million [18]. However, its varied presentations are often not appreciated, and it is probably under-diagnosed. Several reports describe frequent misdiagnoses [5, 7, 10]. Initial diagnostic accuracy was only 19% in one study [10] and 10% in another [7]. The most common misdiagnoses included eye strain or eye irritation from dryness, conjunctivitis, or keratitis. These diagnostic errors contribute to delays between onset and diagnosis that may span many years [4, 7, 10, 11, 19-22]. Although improved awareness in recent years has shortened these delays, more than half of all affected individuals typically require more than a year to reach a diagnosis [21]. The challenges in diagnosing BSP are important to address, because treatments are available.

Our understanding of BSP is based mostly on case reports and case series from expert centers. Some of these focused on idiopathic cases, while others included cases with known causes. Some focused strictly on motor features, while others focused on non-motor features. Methods of ascertainment and assessment varied considerably. The purpose of the current study was to provide a comprehensive description of the clinical features of isolated BSP. First, we provide a summary of 10,324 cases reported in 41 separate manuscripts from the literature. Next, we describe the clinical features of BSP from the Dystonia Coalition (DC), an international multicenter project involving all types of dystonia, 884 of whom had BSP. Finally, we provide details on clinical features associated with spread of dystonia beyond the upper face. By providing a detailed description for a large number of subjects with BSP, we aim to improve clinical recognition and facilitate more timely diagnosis and treatment.

## METHODS

### Systematic Review of Literature

**FIGURE 1 ∣:**
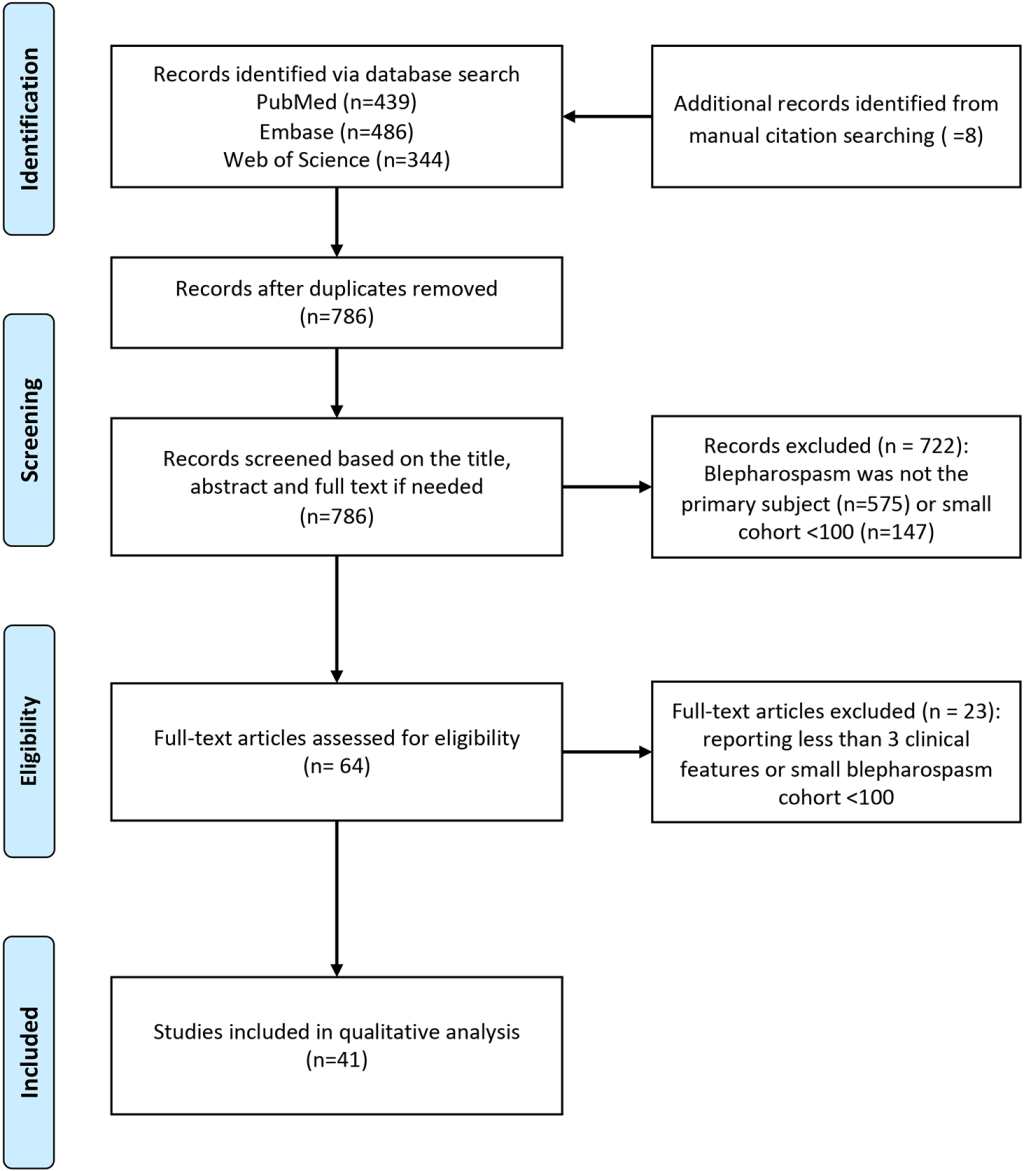
PRISMA flow diagram for literature review.

The Embase (Elsevier), PubMed (National Library of Medicine), and Web of Science: Core Collection (Clarivate Analytics) databases were searched with various combinations of keywords (blepharospasm, eyelid spasm, orbicularis oculi muscle spasm, eyelid contraction, eyelid twitch, cohort studies, longitudinal studies, follow-up studies, prospective studies, retrospective studies) and controlled vocabulary (i.e., MeSH, EMTREE) terms for BSP and cohort studies. The search included cases described as *Meige syndrome*, a term that usually refers to BSP variably combined with involvement of the lower face, jaw, tongue, and sometimes neck [23]. Because of its varied usage, this eponym is not used here. The searches were limited to English language and human studies, with no publication year limit. The authors also reviewed reference lists for potential additional citations. Studies that provided original results for cohorts ≥100 subjects were selected based on the abstract and full text (55 papers for 100–500 cohort and six papers for >500 cohort). Papers that included at least three demographic or clinical features (age at onset, sex, symptoms, triggering or alleviating factors, disease progression, treatment response) were selected for final review. The selection process was completed by two authors independently (LS and HC) and a third author (MH) adjudicated any discrepancies. Forty-one papers including 10,324 total cases were included in the final analysis (Figure 1) [5, 7, 9-11, 14, 15, 17, 21, 22, 24-35]. No effort was made to address potential duplicate reporting, except for three reports describing the same cohort [36-38].

### Dystonia Coalition (DC) Cohorts

Data from the DC were collected and analyzed for subjects enrolled across 45 international sites from 2011–2020 [39]. Subjects recruited for the DC had to be diagnosed with isolated dystonia (focal, segmental, multifocal, generalized). Inclusion in the current study required dystonia to be present in the upper face. Exclusion criteria included any evidence that dystonia that was acquired (e. g., medication-related tardive dystonia), functional (psychogenic), or associated with a known or suspected neurodegenerative disease (e. g., Parkinson’s disease). Also excluded were cases with significant medical or neurologic conditions that might preclude proper evaluation. For this study, *focal* BSP was defined as dystonia limited to the upper face (orbicularis oculi and nearby muscles). Additional involvement of the oromandibular region was considered segmental craniofacial dystonia.

The DC database includes several sub-studies [39]. Data for subjects with BSP were collected from the Biorepository Project, the Natural History Project, and the Blepharospasm Diagnosis Project. Each of these projects collected a core dataset for a total of 884 subjects that included demographic features along with the distribution and severity of dystonia according to individual body regions assessed using the Global Dystonia Rating Scale (GDRS) and the Burke-Fahn-Marsden dystonia rating scale (BFM) [40]. The GDRS is a Likert-like scale in which dystonia is rated from 0 (absent) to 10 (maximal severity). The BFM is a 120-point rating scale used to assess the severity of dystonia in nine body regions taking into account the severity and frequency of movements with a higher score indicative of greater impairment. A subgroup of 386 subjects in the Natural History Project also completed the Beck Depression Inventory II scale (BDI), and Leibowitz Social Anxiety scale (LSAS). A subgroup of 155 subjects in the Blepharospasm Diagnosis Project completed a survey of subjective symptoms such as light sensitivity, eye discomfort, and the presence of alleviating maneuvers. For the DC, recruitment of the same subject more than once was prevented by assigning all subjects a unique identifier and by conducting DNA fingerprinting on blood samples [39].

The study was approved by the IRBs of all participating clinical sites. All participants gave written consent for participation following the principles of the Declaration of Helsinki. The Emory University IRB and the National Institute of Health IRB also approved all procedures involving human participants.

### Statistical Analysis

Because of the different methods and different types of data collected, analyses were completed separately for the literature review and DC cohorts. Results are given as average values ± standard deviations. Descriptive analyses for demographic and clinical characteristics were completed with two sample t-tests for continuous variables and chi-square tests for categorical variables, with *p* < 0.05 considered statistically significant.

For the DC cohort, we also compared BSP cases with onset of dystonia in the upper face to BSP cases with onset elsewhere. The DC cohort was also used to determine factors associated with spread of BSP beyond the face. Multivariate logistic regression was performed to estimate associations between spread of dystonia from the upper face and specific characteristics. Presence of dystonia elsewhere was determined by GDRS>0 in another body region. Demographic and clinical features of interest in the regression analysis were age at onset, dystonia duration, gender, family history, GDRS and BFM scores for upper face, BDI total score, and LSAS total score. A small amount of missing data was identified, but did not vary by exposure or outcome, and was treated as missing at random. All data analysis was performed with SAS version 9.4.

## RESULTS

### Literature Review

#### Demographics and Risk Factors

**FIGURE 2 ∣:**
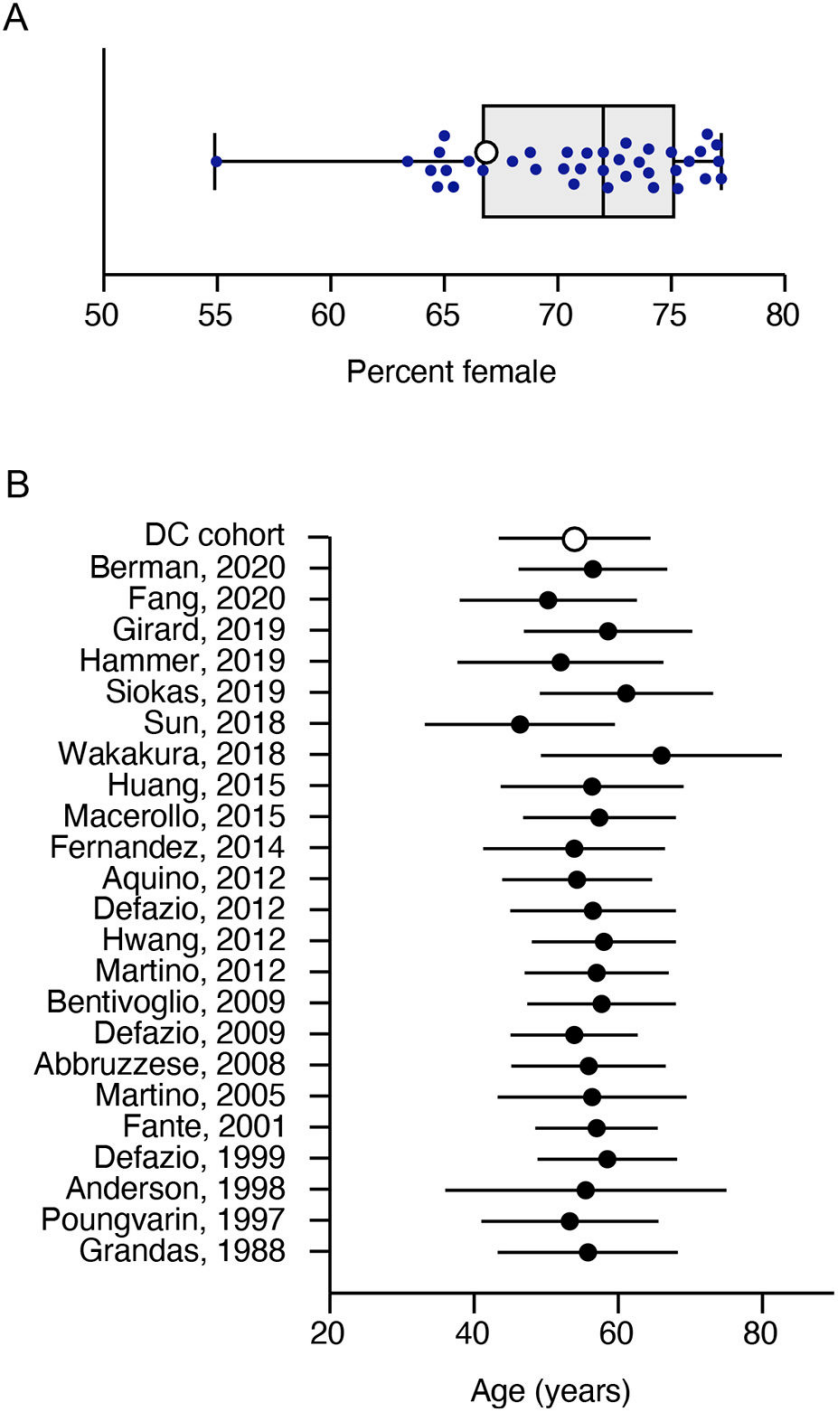
Sex and Age at Onset. **(A)** shows the percentage of females for all publications reaching criteria for inclusion in the literature review. The sex ratio in each study is represented by a filled circle. The open circle shows the sex ratio in the Dystonia Coalition cohort. The box shows the interquartile range of all studies, with the bar in the middle showing the median. Error bars show the full spread of data across all studies. **(B)** shows the mean age at onset in blepharospasm. This plot shows the average (filled circles) and standard deviation (error bars) for all publications reaching criteria for inclusion in the literature review. It also shows the average (open circle) and standard deviation for the Dystonia Coalition cohort.

Among 41 papers considered, 38 reported more females than males, with an overall weighted average of 71% female (Figure 2A). Twenty-one reported median age at onset in 5–6th decade, with an overall weighted average of 56 ± 6 years (Figure 2B). Six reported diagnosis was significantly delayed; two of these revealed that only about half of the patients received the correct diagnosis within one year [4, 10]. The median time from onset to diagnosis was 2 years in one study [22] and the weighted mean from three other studies was 46 ± 68 months [7, 11, 21].

Several studies also addressed potential risk factors for developing BSP including prior eye disease, eye surgery, trauma, stressful event, psychotropic and antiemetic use and white-collar occupation [4, 8-10, 24, 26, 29, 32, 41, 42]. Coffee was reported as potential protective factor in an Italian cohort [41, 43].

#### Presenting Features

Six reports described presenting symptoms of BSP [4, 5, 7, 10, 24, 26]. Increased blinking was the most common (51.9%), followed by ocular pain or soreness (38.7%), photophobia (35.5%), difficulty with eye opening (23.9%) and dry eyes (10.7%). One paper reported the median time to develop BSP following onset of initial symptoms was 12 months for increased blinking, 12 months for difficulty opening eyes, 11 months for dry eyes, and 6 months for photophobia [7]. Although BSP is defined as bilaterally symmetric disorder, three studies reported unilateral onset in 20%, [24] 26%, [44] or 62% [5] of cases.

#### Non-Motor Features in Established Cases

Among cases with established diagnoses of BSP, non-motor features were common. A large-scale epidemiological study [9] and one case-control study [33] identified a link between dry eye and BSP. One study performed a Schirmer I-test on 144 BSP patients by placing a filter paper in the inferior conjunctival fornices for 5-min and measuring the length of wetting [11]. In this study, 86.8% had Schirmer I-test values < 15 mm and 76.4% < 10 = mm consistent with dry eye syndrome. Anxiety (34%), depression (21%), insomnia (11%) and panic attacks (10%) were reported in three cohort studies [4, 7, 8]. The presence of non-motor symptoms did not correlate with severity of motor features [10]. Sensory tricks were reported in four cohort studies [4, 5, 8, 24]. The most common trick in one paper involved touching above the eyes (30%), followed by singing (26%) and talking (25%) [4]. Three papers reported improvement of symptoms with sleep and rest [5, 8,24]. One paper noted sleep benefit and diurnal variations of symptoms in 81% of cases [5].

#### Progression

BSP is considered a chronic disorder and patients often report symptoms worsen over time. BSP also spreads to other parts of the body more frequently than dystonia that starts in other body regions [14, 15, 17, 27, 44, 45]. Most of the spread occurs in the first 5 years. Previous head trauma with loss of consciousness, age at onset and female sex were associated with an increased risk of spread in an Italian cohort [27]. Other factors such as age at onset and alcohol responsiveness have been associated with higher risk of spread [17]. One study suggested that a genetic variant of *TOR1A* may be associated with spread [31]. Spontaneous remissions were reported in three studies with frequencies of 1%, [44] 5%, [24] or 11% [46]. However, the majority of cases experienced recurrence after 1 month to 40 years [24, 44].

### DC Cohort

#### Demographics

Among all 884 cases with BSP, the average age was 64 ± 11, the average age at onset was 49 ± 14, and 67% were female. Only 36% of the 884 cases had isolated focal BSP at onset (defined as dystonia limited to the orbicularis oculi and nearby muscles of upper face), and dystonia remained limited to the face in only 18%. The remainder of BSP cases had dystonia onset in other body regions. Table 1 provides a cross-sectional comparison of demographic and clinical features for cases who had onset in the upper face versus onset in other body regions. Those with onset in the upper face were slightly older with shorter durations of illness than those with onset elsewhere, although the magnitude of the difference was small. BFM and GDRS scores showed that those with onset in the upper face had more severe dystonia in the upper face but less severe dystonia overall than those with onset elsewhere.

#### Specific Motor Features

**FIGURE 3 ∣:**
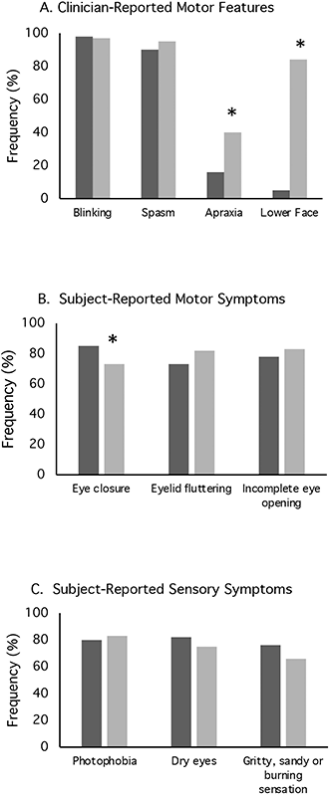
Motor and non-motor features of blepharospasm. These plots illustrate **(A)** clinician reported motor features, **(B)** subject reported motor features, and **(C)** subject reported sensory symptoms for both focal blepharospasm (dark bars) and blepharospasm (light bars) with subsequent spread of dystonia to other body regions. These data were derived from the subgroup of 155 subjects who participated in a blepharospasm rating scales project.

The precise motor features of BSP were evaluated in more detail in a subset of 131 cases participating in a sub-project aimed at assessing these features. Clinician-rated assessments revealed subjects most commonly had mixed features of excessive blinking, periocular spasm, and apraxia of eyelid opening. Of the 131 cases, excessive blinking was reported for 98%, periocular spasm for 93%, and apraxia of eyelid opening for 29% (Figure 3A). One case had spasm without excessive blinking and eight cases had excessive blinking without spasm. None had isolated apraxia of eyelid opening. Associated lower face dystonia was reported for 51%. Subjects commonly reported mixed motor symptoms including involuntary eye closure (79%), incomplete eye opening (58%) and eyelid fluttering (78%) (Figure 3B).

#### Non-Motor Features

Approximately half of the entire cohort of 884 cases reported a sensory trick lessened the severity of their dystonia. Among the 386 subjects in a sub-study that completed scales assessing psychiatric features, social anxiety was present in 40%, as indicated by LSAS scores greater than 30 points. Depression was present in 24%, as indicated by BDI-II scores greater than 13 points. For the subset of 151 subjects in the BSP scales subproject, patients filled out a questionnaire relating to sensory symptoms (Figure 3C) that revealed a high frequency of photophobia (82%), dry eyes (78%), or a gritty, sandy or burning sensation (70%).

#### Progression

Detailed information regarding the spread of dystonia beyond the upper face was available for a subgroup of 224 subjects who participated in a sub-study evaluating natural history (Table 2). Among 224 cases presenting with focal BSP, 61% experienced spread of dystonia. The most common region for spread was to the lower face (84%), followed by the neck (63%) (Table 2). Duration of dystonia, age at onset, and gender were not predictive of spread. Subjects reporting a family history of dystonia were 6.8-times more likely to have spread (*p* < 0.01). Likewise, increased severity of dystonia in the upper face as measured by GDRS and BFM was associated with spread (*p* < 0.01). Subjects in whom clinicians noted apraxia of eyelid opening (*p* < 0.01) and those with coincident lower facial dystonia (*p* < 0.01) were more likely to report subsequent spread (Figure 3A). Subjects reporting involuntary complete eye closure were 4.8 times more likely to develop spread (*p* = 0.04; Figure 3B). Subjective sensory symptoms were not associated with spread (Figure 3C). Both depression as measured by the BDI (*p* < 0.01) and social anxiety as measured by the LSAS score (*p* = 0.03) were associated with spread.

## DISCUSSION

The incorrect and frequently delayed diagnoses reported in the literature highlight the need for better awareness of the many varied clinical features of BSP. To this end, this study provides a comprehensive summary of the clinical features of BSP. Despite different strategies for inclusion and evaluation, results from the literature review are quite similar to those of the DC cohort. Like other focal dystonias [47, 48], BSP tends to emerge in the 50s. Like most other focal dystonias [47, 48], BSP is more common in females. Though spasm of the orbicularis oculi and surrounding muscles may be the most widely recognized feature of BSP, excessive blinking was the most commonly observed sign by clinicians, and eyelid fluttering was frequently reported by subjects. The presence of apraxia of eyelid opening was less common, although its frequency depends on how it is defined. It was observed in approximately a third of DC cases. Finally, non-motor features were common. Sensory features may be a prominent or presenting problem and include irritating eye sensations such as dryness or a gritty feeling, along with photophobia. Depression and anxiety were also common.

The strengths of this study include the use of two independent cohorts, both of which were very large. The literature review included 10,324 cases taken from 41 reports from many parts of the world. The DC cohort is the largest single cohort to be reported, with 884 cases recruited in a multi-center design with 45 centers internationally. The main weakness of this study is inability to directly compare the two cohorts, because of the different methods and designs employed. Another weakness of this study is over-representation of white individuals in the DC cohort, although the results appear to be similar to cohorts of predominantly non-white individuals, such as those from Asia. Another weakness is the lack of information regarding treatment strategies or treatment outcomes, which were not collected for the DC cohort and often not presented for previously published cohorts. The final weakness is the lack of any associated biological measure such as genetics, imaging, or physiology. Unfortunately, the available genes account for <1% of most BSP cases, routine clinical imaging studies rarely reveal any consistent abnormalities, and most centers do not routinely conduct physiological studies. This omission points to the need for additional studies of relevant biomarkers.

Although the clinical features of BSP are quite characteristic, there are several reasons that might account for incorrect or delayed diagnoses. One reason may be that many individuals present first with non-motor symptoms such as irritation of the eyes and/or psychiatric concerns. Eye discomfort with excessive blinking may wrongly suggest allergies or dry eyes, which are very common in this age group. Anxiety and depression may lead to an initial psychiatric diagnosis. The reduction in BSP symptoms when talking while giving the history may wrongly suggest inconsistency or susceptibility to distraction, typical of functional (psychogenic) dystonia. Another reason may be that BSP is relatively uncommon, leading to misdiagnoses of more common disorders. However, common initial misdiagnoses also include myasthenia gravis, which is less common than BSP [10]. One study found myasthenia gravis to be the most common initial misdiagnosis [5], arguing that awareness of BSP may not be as good as awareness of myasthenia gravis.

Regarding the progression of BSP, the results from the DC cohort are consistent with other studies that have revealed a high risk for BSP to spread beyond the upper face [12-17, 27]. Common regions for spread include the lower face, jaw, and neck. Spread to other regions may also occur but appears less common. Factors associated with spread include severity of BSP and family history. Despite the fact that family history is likely to be under-recognized in studies that rely on patient report [49], the association of family history with spread suggests that genetic factors contribute to spread. The current study also revealed that spread was associated with psychiatric features. Whether the psychiatric features are a contributor or consequence of spread cannot be determined from the available data. Similar to prior studies, other sensory features such as dry eyes or photophobia were not associated with spread. Identification of factors related to spread is useful to aid counseling for affected cases.

The importance of early recognition is underscored by the availability of effective treatments. Numerous reviews have been published [50-55]. Several botulinum toxin preparations have proven helpful in the treatment of BSP, with risk/benefit profiles that are similar across brands [50, 51, 53, 54, 56, 57]. Multiple long-term observational studies have demonstrated 68%–89% of individuals show improvement [34, 52, 58-62]. Most studies indicated that mean benefit latency ranged between 4 and 6 days and benefit duration averaged 10 weeks [30, 52, 56, 60], although some describe longer mean response times of 13–16 weeks [22, 25]. Because of their efficacy, botulinum toxins are the treatment of choice for most cases. When botulinum toxins fail, surgical procedures may be offered [63]. Peripheral surgical therapies include orbicularis oculi myectomy, frontalis sling, and differential section of the facial nerve. These procedures were reported to be beneficial in 73–80% of cases [28, 64, 65], although blinded studies with long-term follow-up are not available. Deep brain stimulation also may be offered to patients with BSP. A meta-analysis of 115 cases described in numerous small series suggested deep brain stimulation may be beneficial [66]. However, there is a known bias for publication of positive results, there are no large rigorous blinded studies, and deep brain stimulation has been reported to worsen or trigger BSP in some cases [4, 45]. The optimal surgical approach to BSP remains uncertain. The availability of numerous medical and surgical therapies for BSP emphasizes the need for more rapid accurate diagnosis, which is critical for shortening time from onset of symptoms to treatment in order to alleviate motor symptoms. Treating physicians may also need to address sensory features. For example, artificial tears or other eye lubricants lubrication can be helpful for dry or gritty sensations. Finally, psychiatric features also are common, and they may benefit from direct attention.

## Figures and Tables

**TABLE 1 ∣:** Clinical characteristics of blepharospasm in the Dystonia Coalition cohort.

	Upper face onset N = 320	Other site onset N = 564	*p*-value
Age (years)	64 ± 10	63 ± 12	0.75
Age of Onset (years)	51 ± 13	48 ± 15	<0.01
Duration (years)	12 ± 14	15 ± 15	<0.01
Percent Female	65% (n = 209)	68% (n = 382)	0.46
Areas Affected			
Lower Face, Jaw, Tongue	58% (n = 186)	54% (n = 307)	0.29
Larynx	13% (n = 43)	21% (n = 121)	<0.01
Neck	47% (n = 150)	68% (n = 383)	<0.01
Limbs	16% (n = 36)	17% (n = 29)	0.79
Trunk	3% (n = 9)	8% (n = 42)	<0.01
Sensory Trick	56% (n = 180)	54% (n = 305)	0.53
BFM Severity			
Upper Face	5 ± 2	3 ± 2	<0.01
Total	10 ± 7	11 ± 11	<0.01
GDRS Severity			
Upper Face	5 ± 2	3 ± 3	<0.01
Total	10 ± 7	13 ± 12	<0.01
BDI-II Total Score	9 ± 8	9 ± 8	0.44
LSAS Total Score	29 ± 28	33 ± 29	0.20

This table includes data for the entire cohort of 884 subjects with blepharospasm in the Dystonia Coalition cohort, divided according to those who had onset in the upper face, or those who had onset elsewhere with spread to the upper face. Abbreviations: BDI-II, Beck depression inventory version 2; BFM, Burke-Fahn-Marsden dystonia rating scale; GDRS, global dystonia rating scale; LSAS, Liebowitz social anxiety scale.

**TABLE 2 ∣:** Comparison of blepharospasm cases with and without subsequent spread.

	Focal blepharospasm N = 87	Blepharospasm with subsequent spread N = 137	*p*-value
Age	63 ± 10	63 ± 10	0.91
Age of Onset (years)	51 ± 13	52 ± 13	0.74
Duration (years)	12 ± 13	11 ± 14	0.63
Sex			
Female	70% (n = 61)	68% (n = 93)	0.17
Race			0.17
Asian	7% (n = 6)	2% (n = 3)	
Black	6% (n = 5)	4% (n = 6)	
Other	8% (n = 7)	4% (n = 6)	
White	79% (n = 69)	89% (n = 122)	
Family History of Dystonia	2% (n = 2)	14% (n = 19)	<0.01
Areas Affected			
Lower Face, Jaw, Tongue		84% (n = 115)	
Larynx		16% (n = 22)	
Neck		63% (n = 86)	
Limbs		26% (n = 36)	
Trunk		3% (n = 4)	
Sensory Trick	42% (n = 37)	64% (n = 88)	<0.01
BFM Severity			
Upper Face	4.2 ± 2.5	6.1 ± 1.6	<0.01
Total	4.2 ± 2.5	12.3 ± 8.0	<0.01
GDRS Severity			
Upper Face	4.4 ± 2.1	6.4 ± 1.9	<0.01
Total	4.4 ± 2.1	12.6 ± 6.4	<0.01
BoNT Treatment	82% (n = 72)	79% (n = 108)	0.46
BDI-II Total Score	5.6 ± 7.2	9.0 ± 8.4	<0.01
LSAS Total Score	22.4 ± 26.6	32.0 ± 29.3	0.03

This table includes data for a subset of 224 cases in the Dystonia Coalition database who participated in a sub-study in which information regarding site of origin of dystonia was available. Abbreviations: BDI-II, Beck depression inventory version 2; BFM, Burke-Fahn-Marsden dystonia rating scale; BoNT, Botulinum neurotoxin; GDRS, global dystonia rating scale; LSAS, Liebowitz social anxiety scale.

## Data Availability

The data analyzed in this study is subject to the following licenses/restrictions: Data are being shared upon approval of Dystonia Coalition Executive Committee. Requests to access these datasets should be directed to Dr. Gamze Kilic-Berkmen at dystoniacoalition@emory.edu.
